# Temporal heterogeneity of HER2 expression in metastatic gastric cancer: a case report

**DOI:** 10.1186/s12957-022-02615-0

**Published:** 2022-05-17

**Authors:** Qi Zhang, Tao Yu, Zhicheng Zhao, Shiyao Zhang, Qianpeng Huang, Gang Liu

**Affiliations:** 1 Department of General Surgery, Tianjin Medical University General Hospital, Tianjin, 300052 China; 2 Department of Medical Oncology, Tianjin Medical University General Hospital, Tianjin, 300052 China

**Keywords:** Gastric cancer, Human epidermal growth factor receptor 2, Heterogeneity, Drug resistance

## Abstract

Gastric cancer is a disease with high heterogeneity, and this heterogeneity may result in an uneven distribution of subclones with varied genetic properties at disease locations (spatial heterogeneity) or temporal changes in subclonal composition (temporal heterogeneity). We present the case of a 69-year-old woman with metastatic gastric cancer who presented for axillary lymph node enlargement and underwent axillary lymphadenectomy. Pathological evidence showed human epidermal growth factor receptor 2 (HER2)(3+). Abdominal computed tomography revealed a mass in the gastric body, gastroscopic biopsy showed HER2(3+). After tumor shrinkage by preoperative translational chemotherapy (oxaliplatin, calcium folate, fluorouracil) and targeted therapy (trastuzumab), she had laparoscopic-assisted total gastrectomy. However, HER2 immunohistochemistry was found to be diffusely negative in the surgically removed tissue, and there was no evidence of HER2 amplification in the whole exon sequencing either. After 10 months of trastuzumab treatment, her disease progressed. Although trastuzumab treatment was initially beneficial, the residual HER2-negative subclones may cause tumor recurrence and metastasis due to temporal heterogeneity, as shown in this case.

## Introduction

With approximately 1.09 million new diagnoses and roughly 770,000 patient deaths in 2021, gastric cancer (GC) is the third leading cause of cancer death worldwide and the sixth most frequent malignant tumor [1]. The foundation for treating this exceedingly varied disease is understanding the complicated biological basis of its origin and progression. GC categorization has always been based on histological and morphological characteristics. Lauren developed the classification of GC in 1965, and it is separated into three subtypes: intestinal gastric cancer (IGC, 50%), diffuse gastric cancer (DGC, 33%), and mixed/uncertain (17%) [2]. The enormous complexity of GC, however, cannot be explained solely by histological classification. The TCGA research network identified four GC subtypes based on the large amount of patient data collected from around the world and the unsupervised and integrated clustering of molecular data: tumors positive for Epstein-Barr virus (EBV+), tumors showing microsatellite instability (MSI), genomically stable (GS) tumors, and cases exhibiting chromosomal instability (CIN) [3]. With the defining of new genomic categories and the finding of biomarkers that may predict responsiveness to novel targeted treatments, the molecular mechanisms of GC pathogenesis have gained a better understanding during the last decade. Trastuzumab, an anti-human epidermal growth factor receptor 2 (HER2) antibody, has been approved as the first treatment for late-stage GC with high HER2 expression.

GC is a malignant tumor with a wide range of heterogeneity. Tumor heterogeneity can be found not only among patients with the same type of malignant tumor (intertumor heterogeneity), but also within a single tumor (intratumor heterogeneity) [4]. Intratumor heterogeneity refers to genetic and epigenetic changes in time and space between various subpopulations of tumor cells inside the same tumor [5]. Next-generation sequencing (NGS) can reveal subclones with diverse genetic and epigenetic features in various locations of a single tumor [6]. Intratumor heterogeneity has been revealed in multiple investigations at the genetic, histological, and phenotypic levels. Furthermore, intratumor heterogeneity in GC appears to have harmed the response to HER2-targeted therapies [7]. We present an instance of metastatic gastric cancer with HER2 expression that was temporally heterogeneous during treatment.

## Case presentation

**Fig. 1:**
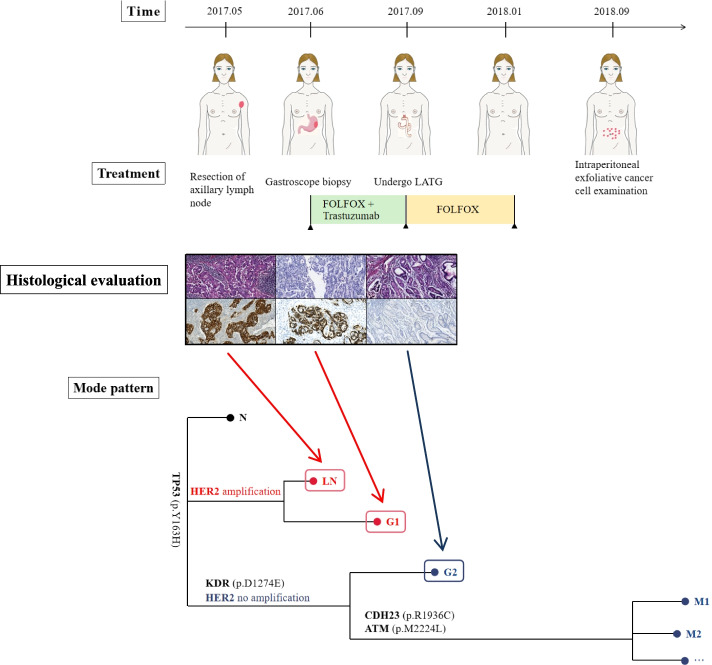
Clinical course over time, including treatments, histological evaluation, pattern, and timing of disease progression

We report a case of HER2 expression temporal heterogeneity in a 69-year-old woman with metastatic gastric cancer. This patient was admitted to the hospital with left axillary lymph node enlargement and underwent axillary lymph node excision. Pathological findings revealed metastatic adenocarcinoma, HER2 (3+), and gene testing verified the presence of HER2 amplification in axillary lymph nodes, prompting a systemic examination to look for the primary tumor, and abdominal computed tomography (CT) revealed a mass in the gastric body. Gastroscopy biopsy revealed gastric adenocarcinoma, HER2 (2+), during the investigation. FOLFOX (oxaliplatin, calcium folate, fluorouracil) + trastuzumab was started 2 months after axillary lymph node excision, and it was well tolerated. She completed three rounds of treatment, followed by a positron emission tomography (PET) CT scan, which revealed that the original lesion had shrunk. Figure 1 depicts the progression of the clinical treatment.

After three sessions of translational therapeutics, a laparoscopic-assisted total gastrectomy (LATG, D2) was successfully done as a curative procedure for an esophageal-jejunum R-Y anastomosis, with no peritoneal metastases discovered. In the stomach body, the removed specimen revealed a 5.5-cm nodular tumor (Borrmann I). Adenocarcinoma penetrating below the serous membrane was found in the histological investigation, with metastases in 7 of the 20 regional lymph nodes. There was no evidence of nerve or vascular invasion (pT3 pN3a [7/20] pM1; stage IV). Immunohistochemical staining detected HER2 (−), while genetic testing revealed no HER2 amplification. The postoperative course was uneventful, and the patient was successfully discharged.

Considering that no HER2 amplification was found on NGS examination of gastric tumor, FOLFOX was given for 5 courses after surgery. The patients underwent regular reviews after surgery. An abdominal CT scan 10 months after surgery revealed enlarged lymph nodes. Subsequently, the patient developed ascites, positive exfoliated cancer cells in the abdominal cavity, and NGS indicated that the tumor cells originated in the stomach without HER2 amplification.

The patient’s condition quickly deteriorated, and she died 2 months after peritoneal metastases were discovered.

## Discussion

In this presentation, we found that a patient with metastatic GC who received trastuzumab plus synchronous chemotherapy successfully resected the main tumor site had variations in HER2 status during treatment, demonstrating that temporal heterogeneity resulted in HER2-negative subclones gaining a growth advantage, leading to recurrence and metastasis.

The anti-HER2 monoclonal antibody trastuzumab combined with conventional chemotherapy enhanced response rates and survival outcomes in GC patients with HER2 amplification. Unfortunately, almost half of the patients do not respond to combination therapy, indicating that primary resistance is present [8]. At the same time, acquired resistance frequently restricts the time it takes for these therapies to work.

Primary resistance to important HER2-targeted medications may be caused by genomic changes in the RTK pathway, such as EGFR FGFR2 MET and KRAS amplification [3]. In vitro in HER2-amplified cell line models, amplifications of cell cycle-related genes such as CCNE1, CDK6, PI3K mutations and MET amplification have shown resistance to HER2 medicines, according to recent research [9]. A study looked at genomic changes in 37 individuals treated with trastuzumab, including EGFR/MET/KRAS/PI3K/PTEN mutations and EGFR/MET/KRAS amplification (17 responders and 20 patients with primary resistance). Panel changes were shown to be much more common in drug-resistant patients (11/20, 55%) than in sensitive patients, and PATIENTS with HER2 IHC (2+) tumors had significantly longer median PFS and OS than those without panel changes [10].

HER2 expression was eliminated in 3 of 7 patients with trastuzumab biopsies before treatment and in the analysis of the same specimen after treatment [11], as has been observed in several cases of acquired resistance to HER2-targeted therapy. Only 6 patients’ HER2 status was stable in a separate examination of 10 patients with primary and paired metastatic samples, while HER2 staining increased or decreased in the remaining 4 patients [12]. This is concerning because many second-line trials do not need a repeat biopsy after first-line trastuzumab to determine HER2 status. For example, the WJOG7112G (T-ACT) test does not require additional HER2 testing, and only 5 of 16 tumors were confirmed to be HER2-positive later. As a result, future second-line trials could boost experimental credibility even further by reassessing HER2 status. In GC, the mechanisms driving acquired resistance to HER2-targeted treatment are poorly understood. One possibility is that members of the HER family, in addition to HER2 expression, can compensate for HER2 blockage. Upregulation of other members of the HER family after HER2-targeted therapy, for example, can activate SRC, resulting in PI3K signaling activation independent of HER2 [13, 14]. Studies revealed that ctDNA testing might detect EGFR and MET amplification during trastuzumab treatment progression [12]. Increased IQGAP1 expression, a scaffolding protein that facilitates HER2 activation and leads to trastuzumab resistance, can also affect the cell surface environment [15]. In medication resistance models, alterations in miRNAs that modulate HER2 and downstream signal conductors like PI3K have also been described [16]. MiRNA regulates the HER2 signaling pathway, which contributes to trastuzumab resistance. Heterodimerization of HER2 with other members of the HER family activates multiple intracellular signaling pathways, one of which being PI3K/AKT. Trastuzumab resistance can be caused by a disruption in the PI3K/AKT pathway. Reducing PTEN in breast cancer cells, for example, causes trastuzumab resistance in vitro and in vivo [17]. The inhibitory effect of trastuzumab on GC cell proliferation was dramatically improved by transfection of miRNA-125A precursor [18]. In breast cancer, MiRNA-199b-5p has been found to improve trastuzumab’s prevention of cell migration and clonal desiccation by blocking HER2 [19]. Trastuzumab-mediated proliferation suppression and cell cycle arrest were reversed when miRNA-542-3p was suppressed, whereas LY294002, a PI3K inhibitor, restored trastuzumab’s effect [20].

## Conclusions

In conclusion, trastuzumab is a successful treatment for GC in many patients. When utilizing trastuzumab to treat unresectable GC, however, the heterogeneity of cancer cells in each patient should be taken into account; not all cancer cells are HER2-positive, and residual HER2-negative cancer cells are still at risk. HER2 status should be reassessed as the disease progresses and changes treatment regimens, and trastuzumab resistance requires more investigation.

## Data Availability

All data generated or analyzed during this study are included in this published article.

## Abbreviations

GC: Gastric cancer
HER2: Human epidermal growth factor receptor 2
NGS: Next-generation sequencing
CT: Computed tomography
PET-CT: Positron emission tomography computed tomography
LATG: Laparoscopic-assisted total gastrectomy
